# Look Trout in the Eye: Corneal Biomarkers of Ammonia Stress in Recirculating Aquaculture Systems Treated with TiO_2_ Photoelectrocatalysis

**DOI:** 10.3390/vetsci13040347

**Published:** 2026-04-02

**Authors:** Giorgio Mirra, Gaia Beatrice Maria Bianchi, Chiara Stocchero, Mirko Sergio, Lucia Aidos, Chiara Bazzocchi, Anna Zurlo, Annamaria Costa, Eleonora Buoio, Silvia Clotilde Modina, Giuseppe Radaelli, Daniela Bertotto, Tarek Temraz, Nadia Chérif, Gian Luca Chiarello, Mauro Di Giancamillo, Alessia Di Giancamillo, Chiara Giudice

**Affiliations:** 1 Department of Biomedical Sciences for Health, University of Milan, 20133 Milan, Italy; 2 Department of Veterinary Medicine and Animal Sciences, University of Milan, 26900 Lodi, Italy; gaiabeatrice.bianchi@unimi.it (G.B.M.B.); chiara.stocchero@unimi.it (C.S.); mirko.sergio@unimi.it (M.S.); lucia.aidos@unimi.it (L.A.); chiara.bazzocchi@unimi.it (C.B.); anna.zurlo@unimi.it (A.Z.); annamaria.costa@unimi.it (A.C.); eleonora.buoio@unimi.it (E.B.); silvia.modina@unimi.it (S.C.M.); mauro.digiancamillo@unimi.it (M.D.G.); chiara.giudice@unimi.it (C.G.); 3 Department of Comparative Biomedicine and Food Science, University of Padua, 35020 Legnaro, Italy; giuseppe.radaelli@unipd.it (G.R.); daniela.bertotto@unipd.it (D.B.); 4 Marine Science Department, Suez Canal University, Ismailia 41522, Egypt; tarek.temraz@gu.edu.eg; 5 National Institute of Marine Sciences and Technologies, Salammbo 2025, Tunisia; nadia.cherif@instm.rnrt.tn; 6 Department of Chemistry, University of Milan, 20133 Milan, Italy; gianluca.chiarello@unimi.it

**Keywords:** rainbow trout (*Oncorhynchus mykiss*), recirculating aquaculture system (RAS), photoelectrocatalytic treatment (PEC), water quality, fish welfare, ocular health

## Abstract

The eye is a sensitive target of sublethal stress in aquaculture-reared fish due to its direct exposure to the aquatic environment. This study tested a photoelectrocatalytic (PEC) water treatment system, integrated into a standard recirculating aquaculture system (RAS), to improve water quality and evaluated ocular health in *Oncorhynchus mykiss* (rainbow trout) reared at 30 kg/m^3^ for 28 days, with particular emphasis on the cornea as an indicator of fish welfare. Ocular analyses focused on the cornea and retina, two anatomically and functionally distinct structures. PEC significantly reduced ammonia levels and modulated nitrate concentrations compared to the control group (CTR), represented by a standard RAS. No differences in growth performance or body condition were observed between groups. Corneal integrity was assessed using optical coherence tomography, histology, and mucous cell staining to evaluate epithelial structure and protective responses. Corneal tissue was examined to detect local oxidative effects through morphological analysis and immunohistochemistry for 8-hydroxy-2′-deoxyguanosine (8-OHdG). Alcian Blu–Periodic Acid–Schiff (AB–PAS) staining did not reveal significant differences in mucin-producing cells among groups. CTR fish exhibited epithelial disruption and increased 8-OHdG immunoreactivity, whereas fish reared in the RAS equipped with the PEC system, ensuring improved water quality, showed preserved corneal architecture despite mild oxidative stress. Molecular analysis of ocular tissues revealed no differential expression of oxidative stress-related genes, such as *GPx1, GR*, or *sod1,* in the two groups. Overall, these findings support the use of the cornea as a sensitive indicator of sublethal environmental stress in farmed fish and suggest that PEC treatment may contribute to improved water quality management and welfare monitoring in intensive aquaculture systems.

## Simple Summary

Fish raised in recirculating aquaculture systems (RASs) are constantly exposed to waterborne stressors that can affect their health and welfare. Because the eye is in direct contact with the surrounding water, it can respond rapidly to changes in environmental quality. In this study, rainbow trouts were reared at high stocking density, and their eye health was examined, with particular attention to the cornea, the outer protective layer of the eye. We compared fish maintained in a standard RAS (CTR) with fish reared in a RAS equipped with an additional photoelectrocatalytic (PEC) water treatment system designed to improve water quality by modulating nitrogenous compounds. Fish kept in the CTR showed signs of corneal damage and altered protective responses, whereas fish reared with the PEC system maintained normal corneal structure, even when mild signs of stress were present. These findings suggest the PEC system improves environmental conditions, and that corneal integrity represents a sensitive and early indicator of fish welfare in intensive aquaculture systems.

## 1. Introduction

Aquaculture has become an indispensable source of protein in the human diet. However, as demand for production rises, concerns regarding environmental sustainability and the welfare of farmed animals are increasingly emerging. One of the main challenges in intensive aquaculture is the fluctuation of water quality parameters, particularly the accumulation of ammonia and nitrogenous compounds resulting from high stocking densities and metabolic waste. Ammonia peaks may occur due to variations in feeding regime and intensity, as well as water flow rate [1].

Elevated ammonia concentrations enhance fish vulnerability to disease, constitute a significant cause of chronic stress in aquaculture, and are strongly linked to adverse effects on health and welfare [2].

Ammonia is a major waterborne stressor, and is particularly toxic in its non-ionized form (NH_3_), which readily diffuses across biological membranes and disrupts cellular homeostasis [3]. In addition to its direct toxicity, elevated ammonia can increase reactive oxygen species (ROS) production, leading to oxidative stress and cellular injury [4].

In teleost fish, ammonia exposure has been shown to induce ROS and perturb antioxidant defenses across tissues [4]. In ocular tissues, excessive ROS can compromise epithelial integrity and barrier function [5].

While the deleterious effects of ammonia on growth performance and internal organs have been widely documented in livestock (chickens [6], pigs [7], cattle [8]) as well as in fish, its impact on ocular tissues has received comparatively less attention. However, the eye may provide a unique window into the health and welfare of farmed fish, and eye damage has been proposed as a key welfare indicator in European aquaculture standards [2].

Specifically, given the absence of eyelids in fish, the cornea and the adjacent scleral conjunctiva are directly exposed to the aquatic environment and highly responsive to subtle changes and may therefore represent a promising source of early welfare indicators. The cornea and sclera are the two components of the outer, fibrous tunic of the eye. The cornea is the outermost interface between the eye and the aquatic environment, functioning both as a transparent refractive surface and a critical barrier against mechanical, chemical, and microbial stressors [9]. Structurally, the cornea consists of a multilayered squamous epithelium, a thick, collagen-rich, avascular stroma, and a thick basement membrane on which a single-layered, thin endothelial layer rests. Corneal stroma is also richly innervated and contains abundant nociceptors [10]. The sclera is the opaque, tough, fibrous posterior part of the outer coat of the eye and is composed of dense, fibrovascular connective tissue. Its anterior part merges with the cornea at the corneoscleral junction. The sclera is covered by conjunctiva, a thin mucous membrane lined by stratified epithelium containing mucus-producing goblet cells. In fish, due to the lack of eyelids, the scleral conjunctiva is continuous with the surrounding skin [11].

The direct exposure of fish corneal and conjunctival tissues to waterborne physical, chemical, and biological stressors makes them sensitive and capable of reflecting subtle changes in environmental conditions, potentially serving as early indicators of fish welfare [10].

Alterations in corneal layers, such as epithelial thinning, stromal disorganization, or delayed wound healing, have been previously related to environmental and toxicological stress in teleosts [12,13] and have been traditionally evaluated through histopathological examination. More recently, Optical Coherence Tomography (OCT) has emerged as a non-destructive, high-resolution imaging technology tool that enables detailed in vivo or ex vivo evaluation of corneal morphology, allowing the detection of subtle alterations, including epithelial thinning or loss, and stromal disorganization that may not be readily evident at gross examination. Although its application in fish research is still limited, OCT has been successfully used to investigate ocular structures in different fish species (zebrafish *Danio rerio* [14], lumpfish *Cyclopterus lumpus* [15]), supporting its potential as a non-destructive technique for the detection of ocular damage.

Photoelectrocatalysis (PEC) has been shown in a recent lab-scale study to be an effective strategy for mitigating ammonia and fluctuations of other nitrogen compounds [16]. By stabilizing nitrogenous compounds, PEC can potentially improve water quality in intensive aquaculture systems. The aim of the present study was therefore to investigate the efficacy of photoelectrocatalysis (PEC) in improving water quality, through the evaluation of ocular health in rainbow trout reared in RASs, with particular emphasis on the cornea as an early and sensitive indicator of environmental stress. Specifically, we investigated corneal alterations in fish maintained in a conventional RAS (CTR) compared with fish reared in a RAS integrated with a PEC water treatment system. An integrated approach combining OCT imaging, histology, and molecular analyses was used to evaluate whether a PEC water purification system, integrated into a RAS, can mitigate fluctuations of nitrogenous compounds under intensive rearing conditions. In this context, the cornea was selected as a target tissue of environmental stress due to its direct exposure to the surrounding water and its high sensitivity to changes in water quality. By integrating OCT, histomorphometric analysis, and immunohistochemical approaches, this study provides a comprehensive evaluation of corneal structural and molecular responses to different water quality management strategies. To align with the objectives of the Special Issue on veterinary morphology and histopathology, we explicitly adopt an integrative morpho-molecular framework that promises to link structural alterations in ocular tissues with early signs of environmental stress and welfare impairment. To the best of our knowledge, this is the first study to combine morphology, advanced ocular imaging, and molecular analyses to explore corneal responses as early biomarkers of welfare and to assess the effectiveness of PEC technology in intensive aquaculture.

## 2. Materials and Methods

### 2.1. Experimental Set-Up

Rainbow trout (*Oncorhynchus mykiss*) were maintained for 28 days in a Recirculating Aquaculture System (RAS) facility (Istituto Sperimentale Italiano Lazzaro Spallanzani, Rivolta d’Adda (CR), Italy). Fish were sourced from a commercial hatchery (Foglio Angelo S.S., Bagolino, Italy) and acclimated for one month before allocation into six experimental tanks (500 L), each operating with an independent recirculating system. Tanks were assigned to either a control group (CTR; *n* = 3), equipped with conventional mechanical and biological filtration and UV disinfection, or an experimental group (PEC; *n* = 3) integrating a photoelectrocatalytic (PEC) water treatment unit.

The PEC unit consisted of a UV-driven electrocatalytic reactor employing titanium dioxide TiO_2_-coated titanium mesh electrodes. TiO_2_ coatings were produced by electrochemical anodization of grade A titanium meshes (39 × 22 cm), rolled into cylindrical elements (4 cm diameter), in an ethylene glycol electrolyte containing 8 M water and 0.2 M hydrofluoric acid, at 30 V for 6 h. The reactor featured a coaxial design with a central UV lamp (35 mm diameter, 900 mm length), surrounded by an inner TiO_2_-coated photoanode (40 mm diameter) and an outer uncoated titanium mesh counter electrode (110 mm diameter), optimizing photoanode irradiation. A constant voltage of 4 V was applied during operation.

Fish, after the acclimatation period, were measured for weight and length (body weight 137.17 g and total length 23.74 cm) and then were stocked at a density of 30 kg/m^3^. Stocking density was adjusted weekly based on fish weight. Fish were maintained under a natural photoperiod, and fed twice daily at 3% of body weight using a commercial diet (Nutria, Skretting Italia S.p.A.,Mozzecane (VR) Italy; following EFSA/FAO recommendations (Commission Regulation 889/2008). Water temperature was kept at 14 °C using a chiller. Fish were weighed weekly to adjust biomass, after light anesthesia in 30 mg/L ethyl 3-aminobenzoate methanesulfonic acid (Sigma-Aldrich, Milan, Italy).

All experimental procedures complied with Italian legislation on animal experimentation (DL 26/2014) and European Directive 2010/63/EU and were approved by the Animal Welfare Body of the University of Milan (authorization no. 62_2023).

### 2.2. Water Parameters

Physical parameters such as water temperature, dissolved oxygen, and pH were monitored daily with a HQ Series Portable Meters (HACH^®^, Linate, Milano, Italy), as previously done by Amini et al. [17]. Chemical parameters like ammonia, nitrites, and nitrates were assessed twice a week using multiparametric tests (HACH^®^, Linate, Milano, Italy) and a Portable Lange DR1900 spectrophotometer (HACH^®^, Linate, Milano, Italy). Titanium presence in water was analyzed by Inductively Coupled Plasma Mass Spectrometry (ICP) according to Wilschefski and Baxter [18].

### 2.3. Fish Sampling

At the beginning of the trial, fish originating from a flow-through rearing system were sampled before experimental allocation (baseline group). At the end of the experimental period (T1; 28 days), fish from each tank (CTR and PEC) were sampled for ocular analyses. Fish were euthanized by overdose of ethyl 3-aminobenzoate methanesulfonic acid (300 mg/L; Sigma-Aldrich, Milan, Italy).

Eyes from eight fish per tank were excised and fixed in 10% buffered formalin. These same fixed eyes were first subjected to ex vivo optical coherence tomography (OCT) as a preliminary screening step and subsequently processed for histological analyses. For OCT and morphological analyses, fish eyes originating from a flow-through system were sampled and used as a descriptive baseline of the situation before the start of the experimental trial (*n* = 8).

For molecular analyses, fresh corneal tissue from three fish per tank was dissected according to Aidos et al. [19], and samples were stored in RNAlater at −80 °C.

### 2.4. Zootechnical Parameters

At the beginning of the trial and T1, fish were weighed and measured for total length. Specific growth rate SGR was calculated following the formula; SGR = 100 × (lnFinalBW-lnInitialBV/days) according to Aidos et al. [20]. The condition factor (K), an index of fish general body conformation, were determined using the formula K = 100 × (BW×TL^−3^), with BW representing the fish’s body weight (g) and TL indicating the total length of the fish (cm), according to Tunçelli and Memiş [21].

### 2.5. Imaging: Optical Coherence Tomography

OCT was performed on eyes (N = 8/tanks, N = 56 in total) previously fixed in 10% buffered formalin and subsequently washed in phosphate-buffered saline (PBS). Before imaging, each eye was positioned to allow optimal alignment with the OCT scanning beam. Corneal structures were visualized using a corneal lens. Following corneal imaging, the globe was carefully opened to expose the posterior chamber, allowing direct visualization of the retina. All OCT examinations were conducted under standardized conditions to ensure consistency among samples.

### 2.6. Morphological Analysis

Following OCT pre-screening, the same formalin-fixed eyes were processed for histological evaluation. Each eye was split into two halves, cutting along the sagittal axis through the center of the cornea and of the optical nerve. The two-resulting samples were routinely processed for histology and embedded in paraffin. Serial microtomic sections (5 µm thick) were obtained and mounted on glass slides. Thus, from each subject, two series of glass parasagittal sections of the central portion of the cornea were obtained.

Hematoxylin and eosin (HE) staining was performed to assess overall ocular morphology and to measure corneal epithelial thickness (N = 8/tanks group, N = 56 in total). Corneal epithelium features were quantified using a semi-quantitative scoring system from 0 to 4:

- 0—Normal integrity: no observable alteration of the epithelial surface;
- 1—Erosion: thinning of the epithelium with loss of superficial layers;
- 2—Superficial ulceration: loss of all epithelial layers with Bowman’s layer exposure;
- 3—Deep ulceration: ulceration with disruption of Bowman’s layer;
- 4—Deep ulceration associated with stromal alteration (edema and neovascularization).

Corneal vessels’ proliferation was also scored semi-quantitatively from 0 to 3:

- 0—Absent;
- 1—Present;
- 2—Marked;
- 3—Marked and associated with pigment deposition.

Corneal epithelium thickness was quantified on digital micrographs using Optika ProView image analysis software (Proview, version 3.7, Optika, Ponteranica, Italy) by taking measurements at three standardized locations along the cornea: the central corneal region and two intermediate points positioned equidistantly between the corneal angle (limbus) and the center of the corneal surface.

Alcian Blue–Periodic Acid–Schiff (AB–PAS) histochemical staining was used to identify and quantify acidic or neutral mucin-producing cells (N = 5/tanks, N = 35 in total). Microtomic sections of the eye of three fish in each group were stained, and a quantitative analysis of muciparous cell density expressed as the number of mucin-producing cells per μm of epithelium was performed using Optika ProView software, considering muciparous cells located at the fornix and in the bulbar conjunctiva and corneoscleral junction.

Immunohistochemical detection of oxidative DNA damage was carried out on three fish in each group using a mouse monoclonal primary antibody against 8-hydroxy-2′-deoxyguanosine (8-OHdG) (clone N45.1, IgG1, Abcam, ab48508, Cambridge, UK) on three subjects of each group (N = 3/tanks, N = 21 in total). Immunohistochemical staining was performed using the Elite ABC KIT system (Vector Laboratories, Inc., Burlingame, CA, USA) as described in Machella et al. [22]. Briefly, after endogenous peroxidase activity and non-specific binding sites were blocked, sections were incubated with primary antibody at 4 °C overnight. After PBS washing, sections were incubated with biotin-conjugated anti-mouse (Dakocytomation), PBS washed, and reacted with peroxidase-labeled avidin-biotin complex (Vector Laboratories, Inc., Burlingame, CA, USA). The immunoreactive sites were visualized using 3.3′-diaminobenzidine tetrahydrochloride (DAB, Sigma, Milan, Italy). To detect structural details, sections were counterstained with Mayer’s haematoxylin. Immunostaining specificity was validated by incubating sections with: (i) PBS instead of the specific primary antibody; (ii) PBS instead of the secondary antibodies. The results of these controls were negative (i.e., staining was abolished).

### 2.7. Molecular Analysis

#### 2.7.1. RNA Extraction and cDNA Synthesis

Total RNA was extracted from corneal tissue using TRIzol reagent (TRI Reagent^®^, Sigma-Aldrich, St. Louis, MO, USA), according to the manufacturer’s instructions. Briefly, corneal samples were homogenized, and the purified RNA was resuspended in 30 μL of RNase-free water. RNA concentration and purity were determined using the NanoReady Touch spectrophotometer (Aurogene, Italy), and RNA quality was assessed by evaluating the A260/A280 absorbance ratio.

Five hundred nanograms of total RNA were reverse-transcribed into complementary DNA (cDNA) using the QuantiTect Reverse Transcription Kit (Qiagen, Hilden, Germany), following the manufacturer’s protocol. A no-reverse transcriptase control was included to verify the absence of genomic DNA contamination. cDNA samples were stored at −80 °C until further analysis.

#### 2.7.2. Gene Expression Analysis

The expression of oxidative response genes (*sod1*, *GPx1*, *GR*) was analyzed using a CFX Duet Real-Time PCR Detection System (Bio-Rad, Hercules, CA, USA) and SYBR Green Supermix (Bio-Rad) as a fluorescent molecule. Primer sequences and the amplification conditions are described in Buoio et al. [23]. Cycle threshold (Ct) values were determined for each sample and normalized using the *EF1α* gene as reference [24,25]. The relative gene expressions of samples collected from PEC groups were calculated using the ΔΔCt method and were compared to samples collected from CTR group, considered as the calibrator.

### 2.8. Statistical Analysis

Statistical analyses of the data were performed with GraphPad Prism software (Version 9.5.0). One-way ANOVA was applied to compare the two experimental groups (CTR and PEC): each tank was considered as an experimental unit. Normality and homogeneity of variances were verified before performing ANOVA. The data are expressed as means ± standard error. Differences between means were considered statistically significant at *p* < 0.05.

## 3. Results

### 3.1. Water Quality Parameters

Water quality parameters measured throughout the experimental period in the CTR and PEC systems show the following results. Ammonia (NH_3_) and nitrate (NO_3_^−^) concentrations differed significantly between treatments, whereas nitrite (NO_2_^−^) levels were slightly lower in PEC-treated tanks. Fish reared in the PEC system experienced a significantly lower mean NH_3_ concentration compared to the CTR (CTR = 1.78 ± 0.20 mg L^−1^ vs. PEC = 0.96 ± 0.20; *p* < 0.05). Conversely, nitrate levels were significantly higher in the PEC tanks than in the CTR tanks (CTR = 53.10 ± 2.14 mg L^−1^ vs. PEC = 61.77 ± 2.14; *p* < 0.05). No significant differences were detected in nitrite concentrations between groups (CTR = 2.84 ± 0.29 vs. PEC = 2.58 ± 0.29). The lower concentration of ammonia, together with the higher concentration of nitrates in the PEC tanks, highlights the superior oxidative capacity of the PEC compared to the biological filter alone.

Titanium was never detected in the water of either group by ICP analysis, demonstrating a high stability of the TiO_2_ coating.

### 3.2. Zootechnical Parameters

**Figure 1:**
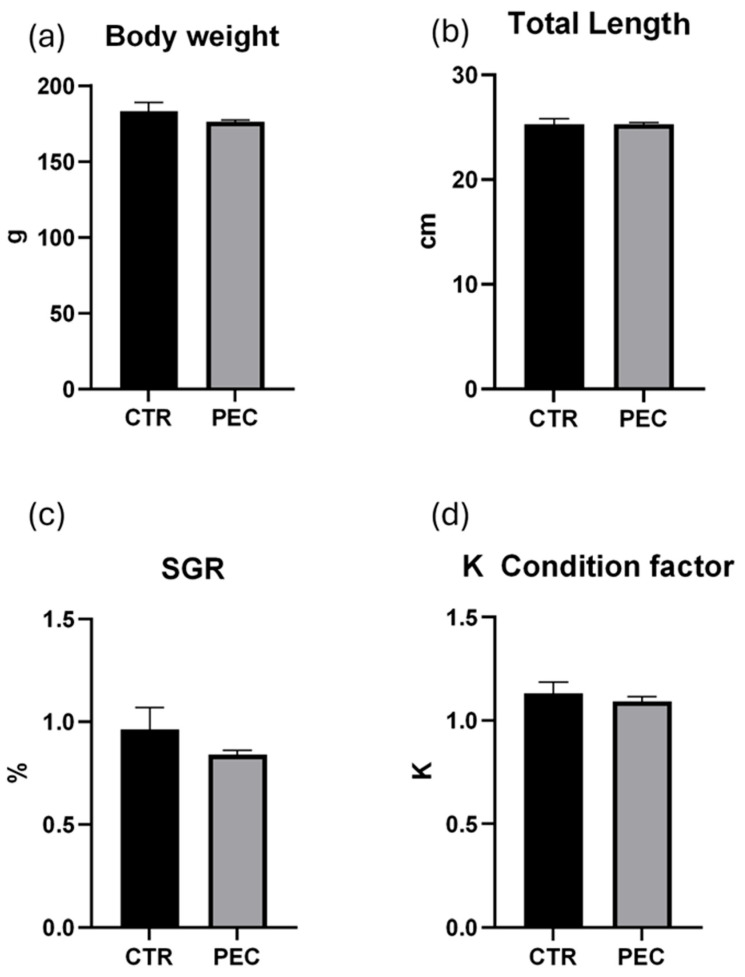
(**a**) Fish growth expressed in g of BW; (**b**) total length expressed in cm of TL; (**c**) SGR expressed in %; (**d**) K condition factor. Error bars indicate the standard error of the mean for each treatment.

Zootechnical performance of rainbow trout reared at high stocking density is shown in Figure 1. Final body weight and total length did not differ significantly between the control and PEC groups (Figure 1a,b). Similarly, no significant differences were observed in specific growth rate (SGR) or condition factor (K) between treatments (Figure 1c,d). Overall, fish growth performance and body condition were comparable between groups throughout the experimental period, indicating that the PEC did not adversely affect zootechnical parameters.

### 3.3. Imaging: Optical Coherence Tomography

**Figure 2:**
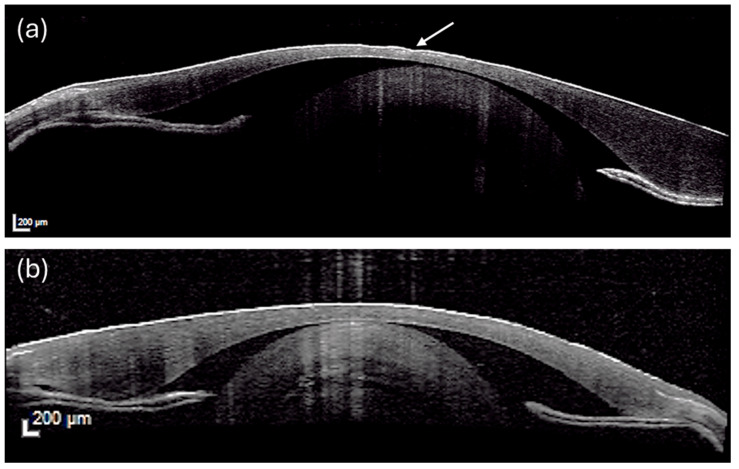
OCT representative images of rainbow trout cornea in CTR (**a**) and PEC (**b**) groups. CTR cornea shows epithelial loss and stromal disorganization (white arrow), while PEC cornea displays intact epithelium and well-organized stroma. Corneal layers and lens are visible. Scale bar 200 µm.

OCT imaging, performed on eight fish per tank as well as on the baseline group, illustrates the structural differences among the experimental groups (Video S1, CTR; and Video S2, PEC). Corneal layers, including epithelium, stroma, and endothelium, were clearly distinguishable, as were the corneal angle, anterior chamber, and lens (crystalline lens). In CTR eyes (Figure 2a), the cornea was characterized by areas of irregular or absent lining epithelium and disorganization of the underlying stromal architecture. In contrast, PEC eyes (Figure 2b) had intact corneal epithelium with uniform thickness and a well-organized stroma, indicative of preserved corneal integrity. Retinal imaging revealed no structural alterations in any of the three groups. All retinal layers were clearly distinguishable and maintained normal stratification.

### 3.4. Morphological Analysis

**Figure 3:**
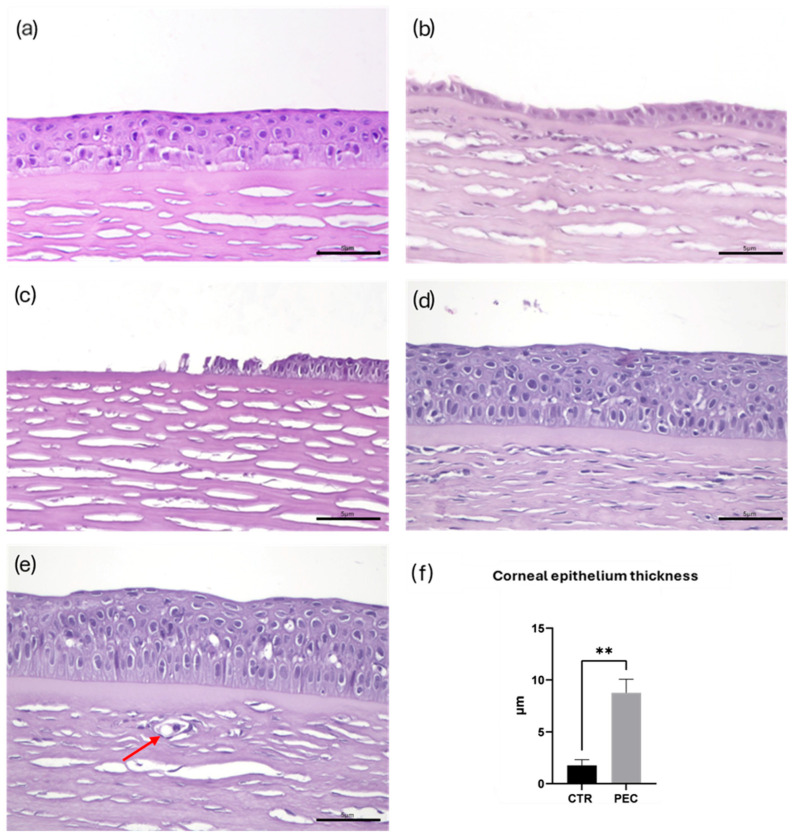
Corneal morphology and histomorphometric analysis. Representative HE-stained corneal sections showing corneal morphology at different experimental conditions: (**a**) baseline fish displaying a well-preserved corneal architecture with an intact epithelial layer; (**b**) control group (CTR): epithelial thinning, characterized by evident epithelial disruption and areas of disepithelialization; (**c**) control group (CTR): epithelial loss (ulceration); (**d**) PEC: largely preserved epithelial structure associated with epithelial thickening; (**e**) PEC: neovascularization (red arrow); (**f**) histomorphometric analysis of corneal epithelium thickness. ** *p* < 0.01; Scale bars 5 µm.

Histological examination of HE-stained slides revealed a well-preserved corneal architecture in the baseline group, in most cases characterized by an intact and continuous epithelial layer (Figure 3a) and a regular, avascular stroma. At the end of the experimental period, the CTR and PEC groups differed in corneal morphology. Control group fish exhibited epithelial disruption with evident and vast areas of thinning (Figure 3b), disepithelialization (Figure 3c), and ulcers. In contrast, fish from the PEC group maintained a more preserved epithelial structure, with fewer and less severe lesions (Figure 3d). In all CTR and most PEC specimens, epithelial lesions were associated with a variable degree of vascular proliferation (Figure 3e). In CTR cases only, severe corneal ulceration, occasionally associated with intense stromal edema, and melanomacrophage stromal infiltration was observed. Globally, histological findings were consistent with OCT results, confirming treatment-related differences in corneal integrity.

At histomorphometric analysis of corneal epithelium, as shown in Figure 3f, epithelium thickness in the PEC group was significantly higher than CTR (*p* < 0.01). Overall, a marked thinning of the corneal epithelium, consistent with epithelial damage (thinning, erosion, and ulceration) was observed in the CTR group, whereas in the PEC group, thickening of the corneal epithelium, possibly related to epithelial proliferation, was observed.

**Figure 4:**
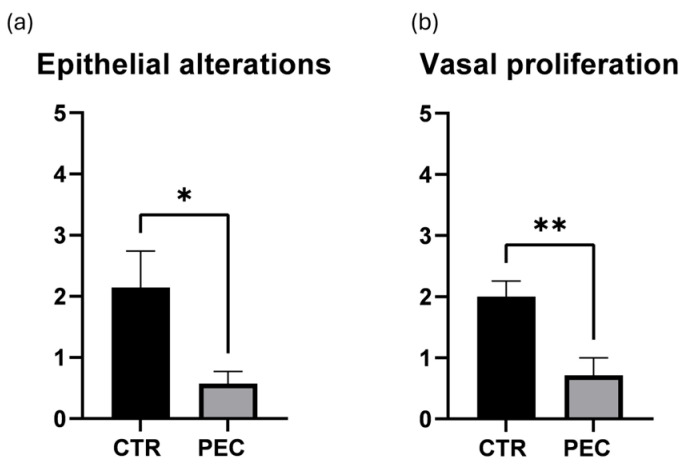
Quantitative assessment of corneal epithelial alterations (**a**) and vascular proliferation (**b**). Data are expressed as mean ± SEM. Statistical significance was determined using one-way ANOVA * *p* < 0.05; ** *p* < 0.01.

As shown in Figure 4a, CTR eyes exhibited a marked increase in epithelial damage (2.14 ± 0.3 vs 0.57± 0.2, *p* < 0.05 vs. PEC). Similarly, vascular proliferation (Figure 4b) was significantly different between CTR eyes and PEC (2.0 ± 0.2 vs. 0.7 ± 0.2, respectively, *p* < 0.01 vs. CTR).

**Figure 5:**
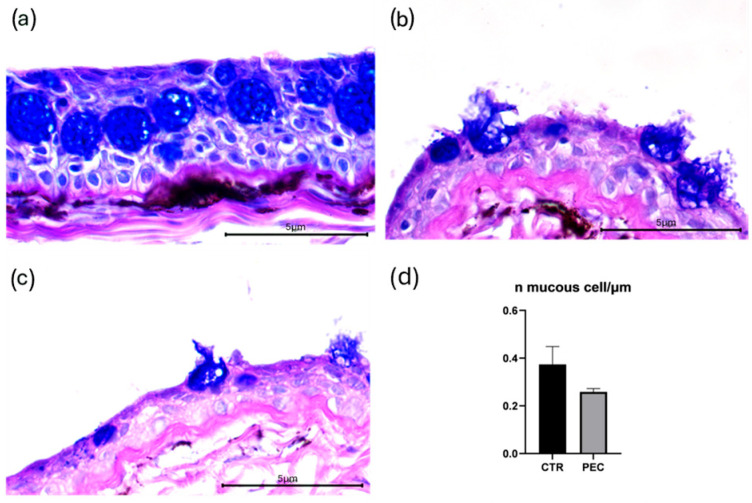
Representative AB–PAS-stained corneal sections showing mucin-producing cells in (**a**) baseline fish, (**b**) CTR at T1, and (**c**) PEC at T1. Acidic mucins are highlighted by blue staining. (**d**) Quantitative analysis of muciparous cell density expressed as the number of mucin-producing cells per μm of epithelium. No significant differences were detected among groups (*p* > 0.05). Error bars indicate the standard error of the mean for each treatment. Scale bars 5 µm.

In all experimental conditions, mucous secretion was predominantly acidic, as indicated by the blue staining observed at the corneoscleral junction and at the bulbar conjunctiva (Figure 5a–c). AB–PAS staining did not reveal significant differences among groups (Figure 5a–c), but an association with melanomacrofages, prevalent in CTR cases, was observed. Quantitative analysis confirmed the absence of significant variations in both the number of muciparous cells and their secretory activity between CTR and PEC groups (Figure 5d).

**Figure 6:**
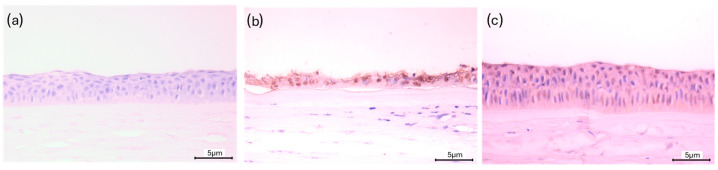
Representative immunohistochemical staining for 8-hydroxy-2′-deoxyguanosine (8-OHdG) in corneal sections of (**a**) baseline fish, (**b**) CTR at T1, and (**c**) PEC at T1. Scale bars 5 µm.

Immunohistochemical analysis using the 8-OHdG antibody revealed the presence of oxidative DNA damage in both experimental groups at the end of the trial. In contrast, no evident 8-OHdG immunoreactivity was observed in corneal tissues in the baseline group (Figure 6a). Quantitative assessment in the CTR was frequently limited due to epithelial loss, whereas preserved epithelial integrity in the PEC allowed more consistent evaluation. As shown in Figure 6b, when the epithelial layer was still present, epithelial cells from the CTR corneas exhibited a more intense 8-OHdG immunoreactivity compared with those from the PEC (Figure 6c). These findings indicate the occurrence of oxidative stress at the ocular surface.

### 3.5. Molecular Analyses

The expression levels of antioxidant-related genes *GPx1*, *GR*, and *sod1* were quantified to evaluate oxidative stress responses in corneal tissues.

**Figure 7:**
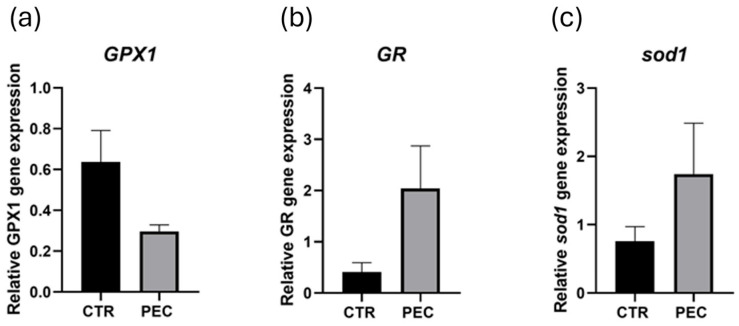
Antioxidant-related gene expression in corneal tissues. Expression levels of (**a**) *GPx1*, (**b**) *GR*, and (**c**) *sod1* were quantified in CTR and PEC groups at the end of the trial. Data are expressed as mean ± SEM.

No statistically significant differences were observed in the expression of *GPx1*, *GR*, and *sod1* between groups. However, *GPx1* showed a decreasing trend in the PEC group compared to CTR, whereas *GR* and *sod1* exhibited an increasing trend in the PEC group (Figure 7).

## 4. Discussion

In this study, an integrated approach combining imaging, morphological, and molecular analyses was employed to evaluate whether a PEC water purification system, integrated into a RAS, can mitigate fluctuations of nitrogenous compounds under intensive rearing conditions. The PEC system successfully reduced ammonia levels. Although nitrate concentrations were higher in the PEC system (61.8 mg L^−1^) than in the control (53.1 mg L^−1^), these levels are below those reported to cause chronic effects in *Oncorhynchus mykiss* in RAS (around 80–100 mg L^−1^ NO_3_-N; [26]), and below the levels at which histological alterations were observed in juvenile turbot (50 mg L^−1^ nitrates showed no changes, while ≥200 mg L^−1^ caused tissue alterations [27]), suggesting that the observed increase is unlikely to impair fish health. In this context, the cornea was selected as a target tissue of environmental stress due to its direct exposure to the surrounding water and its high sensitivity to changes in water quality. Water quality monitoring showed that the PEC system provided a more stable control of nitrogenous compounds compared to the CTR. Although both systems followed similar temporal trends, the PEC group exhibited reduced variability and a more consistent regulation of ammonia, nitrite, and nitrate levels, supporting the effectiveness of photoelectrocatalysis in buffering water quality fluctuations under high-density rearing conditions, confirming previous observations [23]. No titanium was detected in the water, suggesting that the system is safe, and providing information on the chemical stability of the PEC system in aqueous conditions [28].

Despite these differences in water quality, zootechnical parameters did not differ significantly between experimental groups. Growth performance and related indicators remained comparable, suggesting that the 28-day experimental period may not have been sufficient for water quality imbalances to translate into measurable effects at the whole-animal production level. This apparent discrepancy suggests that moderate fluctuations in nitrogenous compounds may not immediately translate into production-level consequences, especially in a species known for tolerance to high stocking densities (up to 100 kg/m^3^) [29]. Rather than reflecting the absence of stress, the lack of growth differences likely indicates that sublethal environmental challenges first manifest at the tissue level before impacting whole-animal performance. In this context, ocular tissues, particularly the cornea, appeared as a highly responsive indicator of environmental variability. The main advantage of using OCT is the acquisition of microscopic images of the entire unprocessed eye. These images can then be easily correlated with histological findings. On the contrary, fixation can introduce artifacts, and measurements obtained on these samples cannot be translated in vivo as such. However, when comparing tissues that underwent the same fixation process, OCT can provide valuable information. Even if OCT data help assess corneal alterations and provide comprehensive visualization of the cornea, its use in farmed fish is currently restricted to research studies. This limitation stems from the high cost of the instrument and the challenges associated with replicating analyses on the same individual. However, OCT identified epithelial thinning and areas of epithelial loss in the cornea of control fish, indicating compromised ocular surface integrity. Histological examination confirmed these findings: CTR corneas exhibited disepithelialization, whereas PEC corneas largely preserved normal architecture. These morphological changes were further quantified by histomorphometric analysis, which showed significant corneal epithelial thinning in CTR fish versus relative preservation or even thickening in PEC fish. Such epithelial thickening in PEC fish may represent an adaptive, early defensive response aimed at reinforcing barrier function under mild stress conditions. Teleost fish are known for remarkable regenerative capacity in epithelial tissues [12]. Although most of the literature on corneal injury mechanisms comes from mammalian models, where thinning and ulceration are established responses to chemical or environmental insult [30,31], our findings suggest that teleost corneas may initiate a proliferative defense at low-to-moderate insult levels, such as mild ammonia elevation. In the CTR group, this compensatory proliferation may have been overwhelmed by greater toxicant load, resulting in widespread epithelial thinning, erosions, and ulcerations. Similar patterns have been reported in fish exposed to waterborne contaminants, where epithelial integrity deteriorates before systemic pathology becomes evident [32,33]. The presence of vascular proliferation in both CTR and PEC corneas further corroborates that ocular tissues mount an angiogenic response to local stress, consistent with teleost wound healing dynamics [34]. Unlike the cornea, retinal architecture remained unaffected across treatments. This is congruent with other reports showing that non-ionized ammonia preferentially affects directly exposed tissues before impacting deeper or immune-privileged structures like the retina in juvenile Atlantic halibut [1]. Taken together, these findings support the use of corneal structural alteration as a sensitive indicator of sublethal environmental stress in intensive aquaculture systems. The fact that corneal epithelial alterations preceded changes in growth performance or condition (which remained similar between groups) underscores the value of tissue-specific endpoints for early welfare monitoring. In Nile tilapia, for example, gill histopathology has been proposed as a sensitive stress marker before growth effects manifest [34], a concept that aligns with our corneal observations in rainbow trout. A limitation of the present study is the absence of intermediate sampling points, preventing the assessment of whether corneal epithelial alterations in the CTR group reflect progressive deterioration or a steady-state condition. However, poor water quality-induced damage is likely to develop progressively. Although such alterations are generally considered progressive, overall, the PEC water treatment system appears to reduce fluctuations in nitrogenous compounds, resulting in fewer morphological disruptions in sensitive ocular tissues.

This insight reinforces recommendations for integrating advanced treatment technologies in recirculating aquaculture systems to sustain both water quality and fish welfare.

Mucous production represents an important defense mechanism in fish, contributing to ocular surface barrier function and defense against chemical and microbial insults [35], and environmental stress can influence mucous cell activity and secretion [35]. Mucin-producing cells were evaluated using AB–PAS staining; no significant differences were observed between groups in terms of mucous cell number or staining characteristics, and acidic mucin secretion was maintained across all experimental conditions.

This stability suggests that mucous secretion may represent a constitutive protective mechanism that is preserved under moderate stress levels, whereas structural epithelial alterations are more sensitive indicators of environmental imbalance.

Goblet cells were occasionally associated with melanomacrophages (MMs), which are pigment-containing phagocytic cells involved in innate immunity and the sequestration of debris and oxidative by-products [36,37,38]. The local association of goblet cells with MMs may reflect a coordinated ocular stress and immune response, suggesting that MMs dynamics could be an informative endpoint for future studies exploring corneal and systemic stress in intensive aquaculture.

Immunohistochemical analysis using the 8-OHdG antibody revealed activation of oxidative stress in ocular tissues from both experimental groups. However, stronger labeling was evident in the control group, particularly in areas where the epithelium was preserved, indicating a higher degree of oxidative DNA damage. Notably, 8-OHdG immunoreactivity was absent in the baseline group, confirming that oxidative stress developed during the experimental period and was associated with rearing conditions. These findings are consistent with previous studies on rainbow trout showing that environmental stressors or toxicants increase 8-OHdG levels in rainbow trout, reflecting oxidative DNA damage as a consequence of reactive oxygen species accumulation [39]. The localization of 8-OHdG within structurally compromised tissues further supports the interpretation that oxidative imbalance is mechanistically linked to epithelial deterioration. The detection of 8-OHdG in ocular tissues highlights the sensitivity of the cornea to sublethal oxidative stress, corroborating the utility of this marker as an early indicator of tissue-specific environmental stress. Comparable patterns have been reported in human ocular tissues, where diseased conjunctiva shows more extensive 8-OHdG staining than healthy controls, emphasizing the relevance of oxidative DNA damage in ocular pathology [40].

The molecular analyses of antioxidant-related genes in the cornea were not significant, but revealed a modulation of antioxidant defenses under varying nitrogenous conditions. *GPx1* expression was higher in the CTR group, which had higher ammonia levels and slightly lower nitrate concentrations, whereas *sod1* and *GR* were higher in the PEC group, characterized by lower ammonia and higher nitrate levels. These trends may reflect different adaptive responses to oxidative challenges: *GPx1* potentially responding to acute ammonia-associated stress, whereas *sod1* and *GR* may represent a modulatory response to elevated nitrates. It is important to interpret these results cautiously. However, the comparative design of the present study, where rearing conditions, water source, and recirculation rates were consistent between groups, minimizes the influence of confounding variables and suggests that the observed differences are plausibly associated with the differential dynamics of nitrogenous compounds.

While ammonia is a possible contributor to oxidative stress, other environmental factors not measured in this study, including overall water quality, may also influence corneal oxidative damage. However, our interpretation is supported by morphological and immunohistochemical findings: CTR corneas exhibited epithelial thinning, disepithelialization, and increased 8 OHdG staining, whereas PEC corneas maintained epithelial structure and organization. Comparable responses have been reported in other fish tissues: ammonia exposure induces oxidative stress and modulates sod, *GPx*, and *GR* activities in the liver, gills, and muscle of Nile tilapia [4], whereas nitrate/nitrite exposure can upregulate glutathione-related defenses, reflecting a broader adaptive response to nitrogenous stress in bighead carp [41]. Moreover, in ocular tissues of zebrafish, nitrate/nitrite exposure alters antioxidant systems and is associated with increased ROS, highlighting the sensitivity of eye tissues to nitrogenous compounds [42].

Overall, while these trends suggest a potential contribution of antioxidant pathways to corneal resilience under differing nitrogenous conditions, we cannot exclude the effects of other environmental stressors, and the lack of direct ROS measurements limits definitive conclusions. The biological basis of this divergent antioxidant response remains to be elucidated.

The integration of the PEC system into RASs was associated with improved control of nitrogenous compounds, which may contribute to reduced sublethal stress and improved fish welfare. Furthermore, the cornea emerged as an early-responsive tissue. Therefore, the cornea may represent a valuable tool for the early monitoring of fish health, allowing timely management actions.

A potential limitation of the present study is the absence of a comparison with systems characterized by poorer water quality or non-recirculating conditions. However, the experimental design was focused on comparing a standard RAS with a RAS integrated with PEC to reflect realistic, widely adopted aquaculture practices. This approach allowed us to evaluate whether the PEC system can provide benefits even under already optimized rearing conditions. In particular, the detection of corneal alterations and oxidative stress signals between groups highlights the sensitivity of corneal biomarkers as early indicators of sublethal stress and the biological relevance of improvements in water quality. Future studies including more contrasting rearing conditions could further elucidate the full extent of PEC system benefits.

## 5. Conclusions

This study demonstrates that the cornea is a sensitive and early biomarker of sublethal environmental stress in farmed rainbow trout. By combining optical coherence tomography, histological evaluation, mucous cell assessment, 8-OHdG immunohistochemistry, and antioxidant gene expression analysis, we were able to detect subtle alterations induced by high-density rearing and fluctuations in nitrogenous compounds.

Fish reared in a conventional RAS (CTR) exhibited epithelial thinning, disepithelialization, oxidative DNA damage, and a less coordinated antioxidant gene response, whereas fish maintained in a PEC-equipped RAS showed preserved corneal structure, lower oxidative stress, and a more balanced activation of antioxidant defenses. These findings support the role of PEC (photoelectrocatalysis) in stabilizing water quality and mitigating sublethal stress in intensive aquaculture. Although ammonia is a well-recognized stressor in aquaculture systems, the present results should be interpreted with caution. The observed corneal alterations and oxidative stress signals cannot be attributed exclusively to ammonia, as the current experimental design does not allow for a direct causal interpretation. In particular, measurements do not fully resolve the contribution of the biologically active ammonia fractions. Overall, the findings suggest that high ammonia likely played a role in the observed ocular changes, although other water quality factors may have acted in combination. Future studies with higher temporal resolution and more detailed characterization of nitrogen species will be necessary to disentangle the specific role of ammonia in ocular pathology.

Overall, integrating morpho-molecular indicators at the ocular surface provides a powerful approach for early welfare monitoring before systemic or growth-related impacts occur. The cornea may represent a sensitive tissue for early welfare assessment in RAS and may support future water management strategies in aquaculture.

## Data Availability

Results are contained within the article. The datasets used and/or analyzed during the current study are available from the corresponding authors on reasonable request.

## Supplementary Materials

The following supporting information can be downloaded at: https://www.mdpi.com/article/10.3390/vetsci13040347/s1. Video S1: Representative OCT imaging of corneal structure in rainbow trout reared in the CTR system; Video S2: Representative OCT imaging of corneal structure in rainbow trout reared in the PEC system.

## Abbreviations

The following abbreviations are used in this manuscript:

RAS	Recirculating aquaculture system
PEC	Photoelectrocatalysis
CTR	Control
AB-PAS	Alcian Blue–Periodic Acid–Schiff
8-OHdG	8-hydroxy-2′-deoxyguanosine antibody
*GPx1*	Glutathione peroxidase 1
*GR*	Glutathione reductase
*sod1*	Superoxide dismutase 1
NH_3_	Ammonia
NO_2_^−^	Nitrite
NO_3_^−^	Nitrate
ICP	Inductively Coupled Plasma Mass Spectometry
ROS	Reactive Oxygen species
OCT	Optical coherence tomography
TiO_2_	titanium dioxide
HE	Hematoxylin and eosin
PBS	Phosphate-Buffered Saline
DAB	3.3′-diaminobenzidine tetrahydrochloride
BW	Body weight
TL	Total length
SGR	Specific Growth Rate
K	K Condition factor
MMs	Melanomacrophages

## References

[1] Liakonis K.M., Waagbø R., Foss A., Breck O., Imsland A.K. (2012). Effects of Chronic and Periodic Exposures to Ammonia on the Eye Health in Juvenile Atlantic halibut (*Hippoglossus hippoglossus*). Fish Physiol. Biochem..

[2] Noble C., Abbink W., Alvestad R., Ardó L., Bégout M.-L., Bloecher N., Burgerhout E., Calduch-Giner J., Chivite-Alcalde M., Císař P. (2026). Welfare Indicators for Aquaculture Research: Toolboxes for Five Farmed European Fish Species. Rev. Aquac..

[3] Thorarensen H., Farrell A.P. (2011). The biological requirements for post-smolt Atlantic salmon in closed-containment systems. Aquaculture.

[4] Hegazi M.M., Attia Z.I., Ashour O.A. (2010). Oxidative Stress and Antioxidant Enzymes in Liver and White Muscle of Nile Tilapia Juveniles in Chronic Ammonia Exposure. Aquat. Toxicol..

[5] Sinha A.K., Liew H.J., Diricx M., Blust R., De Boeck G. (2014). Anti-Oxidative Defences Are Modulated Differentially in Three Freshwater Teleosts in Response to Ammonia-Induced Oxidative Stress. PLoS ONE.

[6] Miles D.M., Miller W.W., Branton S.L., Maslin W.R., Lott B.D. (2006). Ocular responses to ammonia in broiler chickens. Avian Dis..

[7] Stombaugh D.P., Teague H.S., Roller W.L. (1969). Effects of Atmospheric Ammonia on the Pig. J. Anim. Sci..

[8] Fitzgerald S.D., Grooms D.L., Scott M.A., Clarke K.R., Rumbeiha W.K. (2006). Acute Anhydrous Ammonia Intoxication in Cattle. J. Vet. Diagn. Invest..

[9] Pereira P., Raimundo J., Canário J., Almeida A., Pacheco M. (2013). Looking at the aquatic contamination through fish eyes–A faithful picture based on metals burden. Mar. Pollut. Bull..

[10] Ashley P.J., Sneddon L.U., McCrohan C.R. (2006). Properties of corneal receptors in a teleost fish. Neurosci. Lett..

[11] Kong W., Cheng G., Cao J., Yu J., Wang X., Xu Z. (2024). Ocular Mucosal Homeostasis of Teleost Fish Provides Insight into the Coevolution Between Microbiome and Mucosal Immunity. Microbiome.

[12] Ikkala K., Stratoulias V., Michon F. (2022). Unilateral Zebrafish Corneal Injury Induces Bilateral Cell Plasticity Supporting Wound Closure. Sci. Rep..

[13] Uppal R.K., Johal M.S., Sharma M.L. (2015). Toxicological effects and recovery of the corneal epithelium in Cyprinus carpio communis Linn. exposed to monocrotophos: An scanning electron microscope study. Vet. Ophthalmol..

[14] Lichtenegger A., Baumann B., Yasuno Y. (2022). Optical Coherence Tomography Is a Promising Tool for Zebrafish-Based Research—A Review. Bioengineering.

[15] Barter K.R., Paradis H., Gendron R.L., Vidal J.A.L., Meruvia-Pastor O. (2022). Novel segmentation algorithm for high-throughput analysis of spectral domain-optical coherence tomography imaging of teleost retinas. Mol. Vis..

[16] Livolsi S., Franz S., Costa A., Buoio E., Bazzocchi C., Bestetti M., Selli E., Chiarello G.L. (2023). Innovative photoelectrocatalytic water remediation system for ammonia abatement. Catal. Today.

[17] Amini A., Remelli A., Mojarad V.A., Achkar M.E., Dell’Anno M., Rossi L., Mirra G., Giancamillo A.D., Scalvini F.G., Tedeschi G. (2025). From cheese whey to single-cell protein production: By-product valorization through acidogenic fermentation and purple phototrophic bacteria. Bioresour. Technol..

[18] Wilschefski S.C., Baxter M.R. (2019). Inductively Coupled Plasma Mass Spectrometry: Introduction to Analytical Aspects. Clin. Biochem. Rev..

[19] Aidos L., Cafiso A., Lopez A., Vasconi M., Valente L.M.P., Bazzocchi C., Di Giancamillo A. (2022). Rearing Environment during the Endogenous Feeding Stage of *Acipenser baerii*. Animals.

[20] Aidos L., Cafiso A., Serra V., Vasconi M., Bertotto D., Bazzocchi C., Radaelli G., Di Giancamillo A. (2020). How Different Stocking Densities Affect Growth and Stress Status of *Acipenser baerii* Early Stage Larvae. Animals.

[21] Tunçelli G., Memiş D. (2024). The effect of swimming activity and feed restriction of rainbow trout (*Oncorhynchus mykiss*) on water quality and fish-plant growth performance in aquaponics. J. Fish Biol..

[22] Machella N., Regoli F., Cambria A., Santella R.M. (2004). Oxidative damage to DNA: An immunohistochemical approach for detection of 7,8-dihydro-8-oxodeoxyguanosine in marine organisms. Mar. Environ. Res..

[23] Buoio E., Cialini C., Cafiso A., Aidos L., Mazzola S.M., Rossi R., Livolsi S., Di Giancamillo A., Moretti V.M., Selli E. (2022). From Photocatalysis to Photo-Electrocatalysis: An Innovative Water Remediation System for Sustainable Fish Farming. Sustainability.

[24] Khansari A.R., Balasch J.C., Vallejos-Vidal E., Parra D., Reyes-López F.E., Tort L. (2018). Comparative Immune- and Stress-Related Transcript Response Induced by Air Exposure and Vibrio anguillarum Bacterin in Rainbow Trout (*Oncorhynchus mykiss*) and Gilthead Seabream (*Sparus aurata*) Mucosal Surfaces. Front. Immunol..

[25] Eissa N., Wang H.-P., Yao H., Shen Z.-G., Shaheen A.A., Abou-ElGheit E.N. (2017). Expression of Hsp70, Igf1, and Three Oxidative Stress Biomarkers in Response to Handling and Salt Treatment at Different Water Temperatures in Yellow Perch, Perca flavescens. Front. Physiol..

[26] Davidson J., Good C., Welsh C., Summerfelt S.T. (2014). Comparing the effects of high vs. low nitrate on the health, performance, and welfare of juvenile rainbow trout *Oncorhynchus mykiss* within water recirculating aquaculture systems. Aquac. Eng..

[27] Yu J., Wang Y., Xiao Y., Li X., Xu X., Zhao H., Wu L., Li J. (2021). Effects of chronic nitrate exposure on the intestinal morphology, immune status, barrier function, and microbiota of juvenile turbot (*Scophthalmus maximus*). Ecotoxicol. Environ. Saf..

[28] Federici G., Shaw B.J., Handy R.D. (2007). Toxicity of titanium dioxide nanoparticles to rainbow trout (*Oncorhynchus mykiss*): Gill injury, oxidative stress, and other physiological effects. Aquat. Toxicol..

[29] Roque d’Orbcastel E., Person-Le Ruyet J., Le Bayon N., Blancheton J.-P. (2009). Comparative growth and welfare in rainbow trout reared in recirculating and flow through rearing systems. Aquac. Eng..

[30] Dua H.S., Ting D.S.J., Al Saadi A., Said D.G. (2020). Chemical eye injury: Pathophysiology, assessment and management. Eye.

[31] Soleimani M., Naderan M. (2020). Management Strategies of Ocular Chemical Burns: Current Perspectives. Clin. Ophthalmol..

[32] Pascoli F., Negrato E., Di Giancamillo A., Bertotto D., Domeneghini C., Simontacchi C., Mutinelli F., Radaelli G. (2011). Evaluation of oxidative stress biomarkers in *Zosterisessor ophiocephalus* from the Venice Lagoon, Italy. Aquat. Toxicol..

[33] Costa R.A., Cardoso J.C., Power D.M. (2017). Evolution of the Angiopoietin-Like Gene Family in Teleosts and Their Role in Skin Regeneration. BMC Evol. Biol..

[34] Abdel-Moneim A.M., El-Saad A.M.A., Hussein H.K., Dekinesh S.I. (2012). Gill Oxidative Stress and Histopathological Biomarkers of Pollution Impacts in Nile Tilapia from Lake Mariut and Lake Edku, Egypt. J. Aquat. Anim. Health.

[35] Kong W., Yang P., Ding G., Cheng G., Xu Z. (2023). Elucidating the dynamic immune responses within the ocular mucosa of rainbow trout (Oncorhynchus mykiss) after infection with Flavobacterium columnare. Front. Immunol..

[36] Bjørgen H., Koppang E.O. (2024). The melano-macrophage: The black leukocyte of fish immunity. Fish Shellfish. Immunol..

[37] Steinel N.C., Bolnick D.I. (2017). Melanomacrophage Centers As a Histological Indicator of Immune Function in Fish and Other Poikilotherms. Front. Immunol..

[38] Agius C., Roberts R.J. (2003). Melano-macrophage centres and their role in fish pathology. J. Fish Dis..

[39] Topal A., Alak G., Ozkaraca M., Yeltekin A.C., Comaklı S., Acıl G., Kokturk M., Atamanalp M. (2017). Neurotoxic responses in brain tissues of rainbow trout exposed to imidacloprid pesticide: Assessment of 8-hydroxy-2-deoxyguanosine activity, oxidative stress and acetylcholinesterase activity. Chemosphere.

[40] Alak G., Ucar A., Parlak V., Yeltekin A.Ç., Taş I.H., Ölmez D., Kocaman E.M., Yılgın M., Atamanalp M., Yanık T. (2017). Assessment of 8-hydroxy-2-deoxyguanosine activity, gene expression and antioxidant enzyme activity on rainbow trout (*Oncorhynchus mykiss*) tissues exposed to biopesticide. Comp. Biochem. Physiol. Part C Toxicol. Pharmacol..

[41] Lin Y., Miao L.-H., Pan W.-J., Huang X., Dengu J.M., Zhang W.-X., Ge X.-P., Liu B., Ren M.-C., Zhou Q.-L. (2018). Effect of nitrite exposure on the antioxidant enzymes and glutathione system in the liver of bighead carp. *Aristichthys nobilis*. Fish Shellfish. Immunol..

[42] Saputra F., Kishida M., Hu S.-Y. (2024). Nitrate and Nitrite Exposure Induces Visual Impairments in Adult Zebrafish. Toxics.

